# Mirodenafil ameliorates skin fibrosis in bleomycin-induced mouse model of systemic sclerosis

**DOI:** 10.1080/19768354.2021.1995486

**Published:** 2021-11-03

**Authors:** Jong Seong Roh, Hoim Jeong, Beomgu Lee, Byung Wook Song, Seung Jin Han, Dong Hyun Sohn, Seung-Geun Lee

**Affiliations:** a Department of Herbal Prescription, College of Korean Medicine, Daegu Haany University, Gyeongsan, Republic of Korea; b Department of Microbiology and Immunology, Pusan National University School of Medicine, Yangsan, Republic of Korea; c Biomedical Research Institute, Pusan National University Hospital, Busan, Republic of Korea; d Division of Rheumatology, Department of Internal Medicine, Pusan National University School of Medicine, Pusan National University Hospital, Busan, Republic of Korea; e Department of Biotechnology, Inje University, Gimhae, Republic of Korea; f Institute of Digital Anti-Aging Healthcare, Inje University, Gimhae, Republic of Korea

**Keywords:** Fibrosis, systemic sclerosis, mirodenafil, cyclic guanosine monophosphate

## Abstract

Systemic sclerosis (SSc) is a chronic autoimmune disease characterized by fibrosis of the skin and internal organs. Despite the recent advances in the pathogenesis and treatment of SSc, effective therapies for fibrosis caused by SSc have not yet been established. In this study, we investigated the potential role of mirodenafil, a potent phosphodiesterase 5 (PDE5) inhibitor, in the treatment of fibrosis in SSc. We used a bleomycin (BLM)-induced SSc mouse model to mimic the typical features of fibrosis in human SSc and examined the dermal thickness to assess the degree of skin fibrosis after staining with hematoxylin and eosin or Masson’s trichrome stains. The effect of mirodenafil on the expression of profibrotic genes was also analyzed by treating fibroblasts with transforming growth factor (TGF)-β and mirodenafil. We showed that mirodenafil ameliorated dermal fibrosis and downregulated the protein levels of fibrosis markers including COL1A1 and α-SMA in the BLM-induced SSc mouse model. Further, using mouse embryonic fibroblasts and human lung fibroblasts, we demonstrated that the expression of collagen and profibrotic genes was reduced by treatment with mirodenafil. Finally, we showed that mirodenafil inhibited TGF-β-induced phosphorylation of Smad2/3 in fibroblasts, which suggested that this drug may ameliorate fibrosis by suppressing the TGF-β/Smad signaling pathway. Our findings suggest that mirodenafil possesses a therapeutic potential for treating fibrosis in SSc.

## Introduction

Systemic sclerosis (SSc) is a chronic, multisystem, rheumatic disease of unknown etiology that results from complex interactions among the autoimmune system, inflammation, vasculopathy, and fibrosis. Microvascular injury followed by defective angiogenesis/vasculogenesis, vascular wall remodeling, and aberrant inflammatory response triggered by autoimmunity are known to be the initial events in the pathogenesis of SSc. These eventually contribute to widespread fibrosis caused by the excessive accumulation of extracellular matrix (ECM), including collagen in the skin and other internal organs, such as the lung (Kim et al. 2019; Sierra-Sepulveda et al. 2019). The activation of fibroblasts leading to their transdifferentiation into proliferative myofibroblasts is considered a key component of the fibrotic process in SSc (van Caam et al. 2018). A large body of evidence indicates that a variety of immune cells, cytokines, growth factors, and molecular pathways endow myofibroblasts with contractile and proliferative properties, thus exerting a profibrotic role in SSc pathogenesis (Korman 2019; Kim et al. 2020; Kim et al. 2021). Extensive fibrosis is considered a hallmark of SSc and is associated with significant morbidity, disability, and mortality. Although significant advances have been made in understanding the pathophysiology of SSc and several ongoing clinical trials have reported promising preliminary results, effective therapies for fibrosis in SSc have not yet been established. Hence, the management of SSc remains a challenge, and there is still a need to discover novel therapeutic agents.

To date, numerous experimental animal models for SSc have been established, among which the bleomycin (BLM)-induced SSc model has been widely used because of its ease of handling and applicability to most mouse strains. BLM is a chemotherapeutic agent; however, it also has a profibrotic effect. Repeated subcutaneous injection of BLM in mice can induce dermal and pulmonary fibrosis resembling SSc (Yamamoto 2010). Since the lung and skin lack BLM hydrolase, which can inactivate BLM, BLM-induced fibrosis usually occurs in these organs (Yamamoto 2010). Fibrosis induced by BLM is mediated by the production of inflammatory and profibrotic cytokines, such as transforming growth factor-β (TGF-β) and reactive oxygen species (ROS), as well as by the direct effect on ECM synthesis through myofibroblast stimulation (Yamamoto 2010). Increased TGF-β levels in BLM administration promotes Smad 2/3 phosphorylation (canonical TGF-β pathway) and enhances the expression of fibrogenic genes, such as *Col1a1* and *α-SMA* in the BLM-induced SSc model.

Phosphodiesterases (PDEs) are enzymes that degrade phosphodiesterase bonds in cyclic nucleotides, such as cyclic adenosine monophosphate and cyclic guanosine monophosphate (cGMP), converting them into their inactive form. The PDE superfamily consists of at least 11 isoforms, namely PDE1–PDE11. Among these isoforms, PDE5 hydrolyzes cGMP and is expressed in various organs and tissues, including the vascular smooth muscle cells, lung, penile corpus cavernosum, brain, kidney, cardiac myocytes, and gastrointestinal tract (Ahmed et al. 2021). Nitric oxide (NO) activates soluble guanylyl cyclase (sGC), which in turn increases cGMP production. cGMP also plays numerous biological roles, including vascular smooth muscle cell relaxation, tissue perfusion, cytoprotection, and the inhibition of inflammation through the activation of protein kinase G (PKG) and regulation of ion channels (Papapetropoulos et al. 2015). Accordingly, several PDE5 inhibitors have been developed and applied for the treatment of erectile dysfunction, benign prostatic hyperplasia, and pulmonary arterial hypertension (PAH) (Papapetropoulos et al. 2015). A growing body of research has suggested that the stimulation of the sGC-cGMP-PKG pathway mitigates fibrosis by suppressing TGF-β (Beyer et al. 2012; Dees et al. 2015; Matei et al. 2018). Thus, it is hypothesized that PDE5 inhibitors would exert an anti-fibrotic effect in SSc by enhancing the production of cGMP. In this study, we reported that mirodenafil, a potent PDE5 inhibitor, ameliorates dermal fibrosis in a BLM-induced SSc mouse model by inhibiting the TGF-β signaling pathway, thereby suppressing the expression of collagen and profibrotic genes.

## Materials and methods

### Reagents and antibodies

Bleomycin was generously donated by Dong-A ST Co., Ltd. (Seoul, Korea), while mirodenafil was generously donated by SK Chemicals Co., Ltd. (Seongnam, Korea). Anti-α-smooth muscle actin (SMA) antibody (MAB1420) was purchased from R&D Systems. Anti-COL1A1 antibody (3G3) was purchased from Santa Cruz Biotechnology. Anti-phospho-Smad2/3 antibody (D27F4), anti-Smad2/3 antibody (D7G7), and anti-glyceraldehyde 3-phosphate dehydrogenase (GAPDH) antibody (2118S) were purchased from Cell Signaling Technology. Anti-β-actin antibody (AC-15) was purchased from Sigma-Aldrich. Recombinant human and mouse TGF-β1 proteins were purchased from R&D Systems.

### SSc mouse model

**Figure 1.**
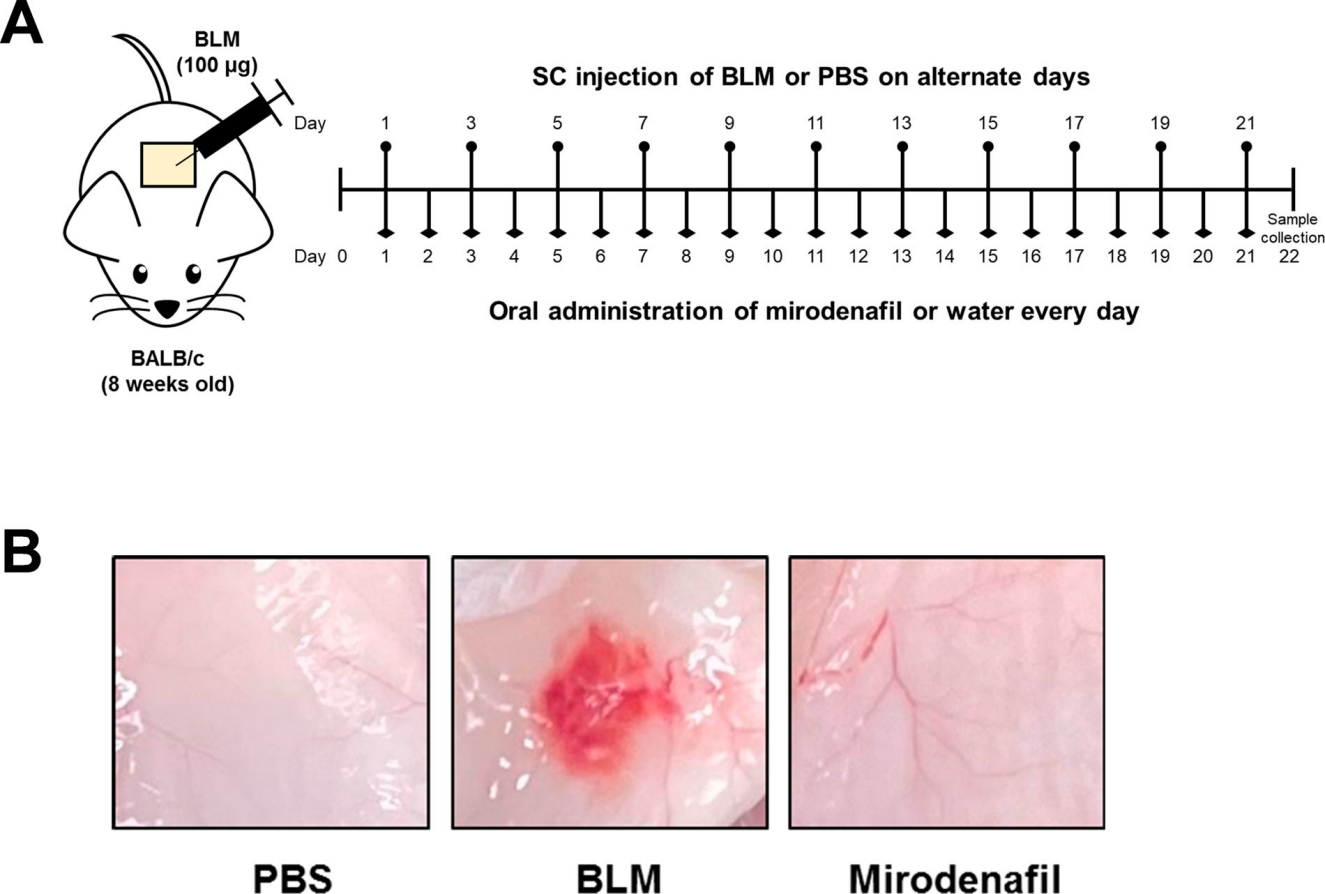
The summary of the systemic sclerosis experimental model. (A) Eight weeks-old male BALB/c mice were subcutaneously injected with either bleomycin (BLM) (100 µg) or phosphate-buffered saline (PBS) on their shaved back on alternate days for 3 weeks. Mirodenafil (5 or 10 mg/kg/day) or distilled water was orally administered every day for 3 weeks. (B) A visible change in the inner surface of the mouse dorsal skin is shown.

All animal experiments were performed under the approval of the Institutional Animal Care and Use Committee of Pusan National University (IACUC #PNU-2020-2685). Male BALB/c mice were purchased from Koatech and housed under specific pathogen-free conditions. The mice were randomly divided into four groups as follows: normal control (NC) group (n=10), BLM group (n=10), mirodenafil 5 mg/kg group (n=10), and mirodenafil 10 mg/kg group (n=10). BLM was dissolved in sterile Dulbecco’s phosphate-buffered saline (DPBS) at a concentration of 1 mg/ml. For the BLM and mirodenafil groups, BLM (100 μl) was injected subcutaneously (SC) on the shaved back of 8 weeks-old mice on alternate days for 3 weeks, while mice received the same volume of DPBS for the NC group. Mirodenafil (5 or 10 mg/kg/day) or distilled water was administered orally together with BLM or DPBS injection. Mice were sacrificed 3 weeks after BLM injection. Skin tissues were fixed in 10% neutral buffered formalin for the histological analysis or processed for RNA or protein extraction (Figure 1).

### Histological analysis

Skin tissues were stained with hematoxylin and eosin (H&E) or Masson’s trichrome stains to measure the dermal thickness. We assessed the dermal thickness from the dermal-epidermal junction to the dermal-subcutaneous fat junction as previously described (Yoshizaki et al. 2010). The thickness was calculated from five randomly selected fields per specimen by two independent examiners. An Olympus BX51 microscope was used to measure the dermal thickness.

### Cell culture

NIH3T3 mouse embryonic fibroblasts were cultured in Dulbecco’s modified Eagle’s medium (DMEM) (Sigma-Aldrich) supplemented with 10% bovine serum (BS). MRC-5 human fetal lung fibroblasts were cultured in minimum essential medium (MEM) (Sigma-Aldrich) supplemented with 10% fetal bovine serum at 37°C in a 5% CO_2_ humidified incubator. NIH3T3 and MRC-5 cells were placed into 6-well culture plates at a density of 3×10^5^ or 2 × 10^5^ cells per well on the day before activation. On the day of activation, media were aspirated and changed to DMEM supplemented with 0.1% BS for NIH3T3 or serum-free MEM for MRC-5. We then treated the cells with mirodenafil for 24 h in the presence of TGF-β1 (10 ng/ml). After treatment, the cells were analyzed for gene and protein expression.

### Quantitative real-time polymerase chain reaction (qPCR)

Total RNA was isolated using an RNA isolation kit (RNeasy Mini Kit, QIAGEN) according to the manufacturer's instructions. Reverse-transcribed cDNA from total RNA was synthesized using a cDNA synthesis kit (SmartGene). Quantitative real-time PCR was performed using SYBR qPCR Master Mix with Low Rox (SmartGene) with the following primers: mouse *α-SMA* (*Acta2*) forward, GTCCCAGACATCAGGGAGTAA; mouse *α-SMA* (*Acta2*) reverse, TCGGATACTTCAGCGTCAGGA; mouse *Col1a1* forward, ACATGTTCAGCTTTGTGGACC; mouse *Col1a1* reverse, TAGGCCATTGTGTATGCAGC; mouse *Ctgf* forward, CTGCAGACTGGAGAAGCAGA; mouse *Ctgf* reverse, GATGCACTTTTTGCCCTTCTT; mouse *Gapdh* forward, TGTGAACGGATTTGGCCGTA; mouse *Gapdh* reverse, ACTGTGCCGTTGAATTTGCC; human *α-SMA* (*ACTA2*) forward, GTGTTGCCCCTGAAGAGCAT; human *α-SMA* (*ACTA2*) reverse, GCTGGGACATTGAAAGTCTCA; human *COL1A1* forward, CCCGGGTTTCAGAGACAACTTC; human *COL1A1* reverse, TCCACATGCTTTATTCCAGCAATC; human *CTGF* forward, GCGTGTGCACCGCCAAAGAT; human *CTGF* reverse, CAGGGCTGGGCAGACGAACG; human *GAPDH* forward, GAAGGCTGGGGCTCATTT; and human *GAPDH* reverse, ATGGTTCACACCCATGACG. Expression data were normalized to the mean of the housekeeping gene *Gapdh* to control the variability in expression levels. The results were analyzed using the 2^-ΔΔCT^ method as previously described (Park et al. 2019).

### Western blot analysis

Cells were lysed in IP lysis buffer (Thermo Fisher Scientific) containing a protease inhibitor cocktail and phosphatase inhibitor cocktail (GenDEPOT). Skin tissues were lysed in sodium dodecyl sulfate (SDS) lysis buffer (1% [w/v] SDS, 10 mM ethylenediaminetetraacetic acid, 50 mM Tris, pH8.0) without a protease inhibitor cocktail and phosphatase inhibitor cocktail. Equal amounts of cell or tissue lysates were resolved on 10% SDS-polyacrylamide gels, transferred to a polyvinylidene fluoride membrane (Millipore), and blocked with 5% nonfat dried milk in a mixture of tris-buffered saline and Tween-20. Thereafter, the membrane was probed with primary and secondary antibodies, and the membrane was developed using a chemiluminescence ECL detection system (TransLab). The signals were detected using an Imagine ABI680 Analyzer (Amersham). Specific bands from western blots were quantified using ImageJ software and normalized against GAPDH as previously described (Woo et al. 2020).

### Statistical analysis

Statistical analysis was performed using GraphPad Prism software (version 9.0; GraphPad Software). Differences among groups were evaluated using Student’s t-test or one-way analysis of variance. All data are presented as mean ± standard deviation, and statistical significance was set at *p*<0.05.

## Results

### Mirodenafil ameliorated dermal fibrosis in BLM-induced SSc mouse model

**Figure 2.**
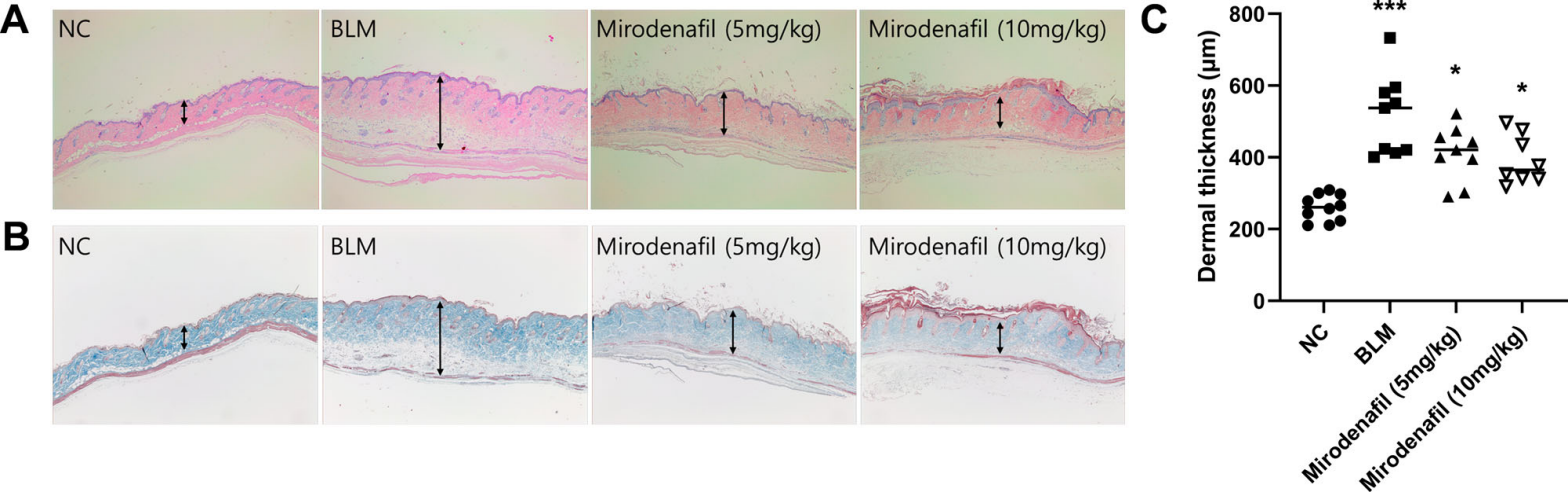
Mirodenafil ameliorated skin fibrosis in BLM-induced systemic sclerosis (SSc) mouse model. Hematoxylin and eosin staining (A) and Masson’s trichrome staining (B) in normal control (NC), BLM, and mirodenafil groups (The original magnification 40×). (C) The dermal thickness of each group (n=8∼10). Data are presented as mean ± standard deviation (SD). *P < 0.05. ***P < 0.001.

**Figure 3.**
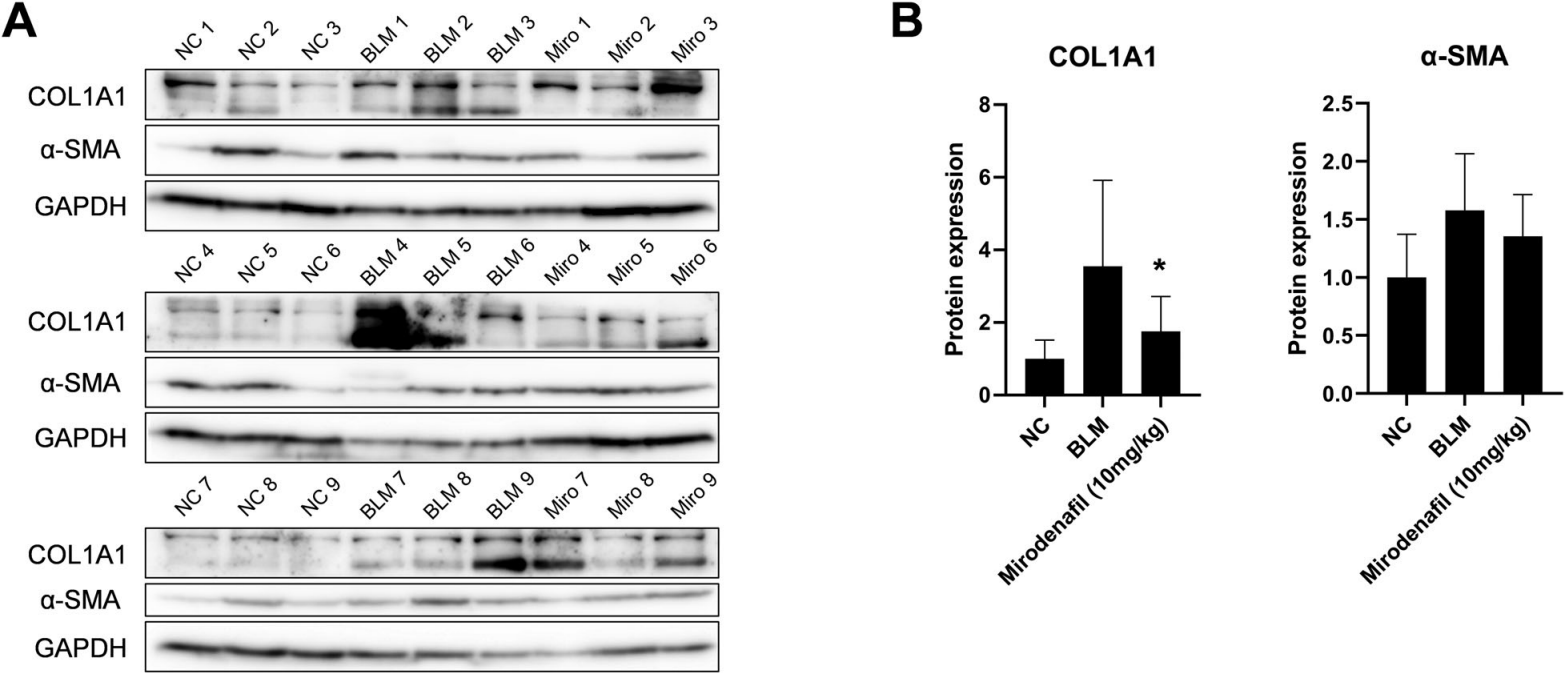
Mirodenafil downregulated the expression of fibrosis markers in BLM-induced SSc mouse model. The protein levels of COL1A1 and α-SMA in the lesioned skin from each group were measured by western blot. Glyceraldehyde 3-phosphate dehydrogenase (GAPDH) was used as loading control. (B) Data from western blot were quantified by densitometry. Data are presented as mean ± SD. *P < 0.05.

To evaluate the effect of mirodenafil on skin fibrosis, BLM-induced SSc mouse models were treated with mirodenafil and dermal thickness of lesioned skin sections were measured. The H&E and Masson’s trichrome staining revealed that the BLM group showed increased dermal thickness and collagen content compared to the PBS-injected normal control (NC) group. On the contrary, the mirodenafil (5 and 10 mg/kg) groups showed significantly decreased dermal thickness and collagen content compared with the BLM group (Figure 2). The dermal thickness was 259.52 μm ± 37.13 (NC), 517.14 μm ± 194.72 (BLM), 411.45 μm ± 76.04 (mirodenafil, 5 mg/kg), and 392.51 μm ± 67.94 (mirodenafil, 10 mg/kg), respectively. In addition, the protein levels of COL1A1 and α-SMA in the lesioned skin were reduced in the mirodenafil groups compared with those in the BLM group (Figure 3). These results suggested that mirodenafil ameliorated dermal fibrosis, probably by suppressing the expression of profibrotic genes.

### Mirodenafil reduced the expression of collagen and profibrotic genes in fibroblasts

**Figure 4.**
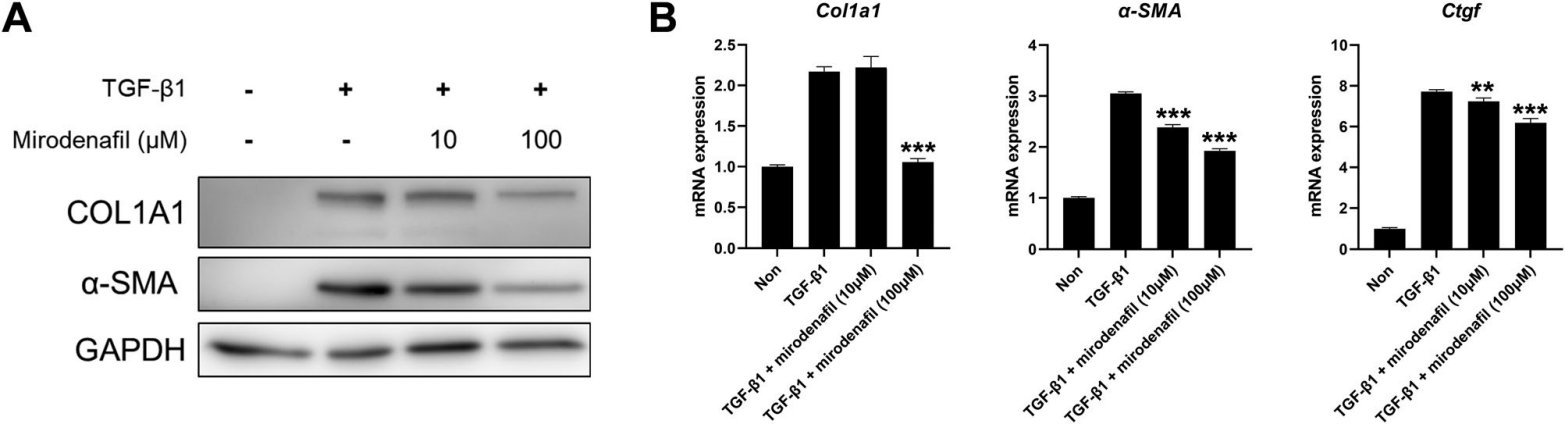
The anti-fibrotic effect of mirodenafil in mouse embryonic fibroblasts. NIH3T3 mouse embryonic fibroblasts were treated with the indicated concentration of mirodenafil and mouse transforming growth factor (TGF)-β1 (10 ng/ml) for 24 hr. The protein levels of COL1A1 and α-SMA were analyzed by western blot (A), and mRNA levels of *Col1a1, α-SMA,* and *Ctgf* were assessed by quantitative real-time polymerase chain reaction (qPCR) (B). GAPDH was used as loading control for western blot and normalization control for qPCR. Data are presented as mean ± SD. **P < 0.01, ***P < 0.001.

To investigate the anti-fibrotic effect of mirodenafil, we stimulated NIH3T3 mouse embryonic fibroblasts with recombinant active TGF-β1 and treated them with different concentrations of mirodenafil. We then examined the protein expression of fibrosis markers, such as COL1A1 and α-SMA. As shown in Figure 4A, the protein levels of COL1A1 and α-SMA were upregulated by treatment with TGF-β1, which was decreased by mirodenafil in a dose-dependent manner. Next, we examined the transcription levels of profibrotic genes, such as *Col1a1, α-SMA*, and *Ctgf.* Similar to protein expression, the mRNA expression of *Col1a1, α-SMA*, and *Ctgf* was induced by TGF-β1, which was also decreased by treatment with mirodenafil (Figure 4B). These results showed that mirodenafil reduced both the mRNA and protein expression of fibrosis markers and profibrotic genes.

**Figure 5.**
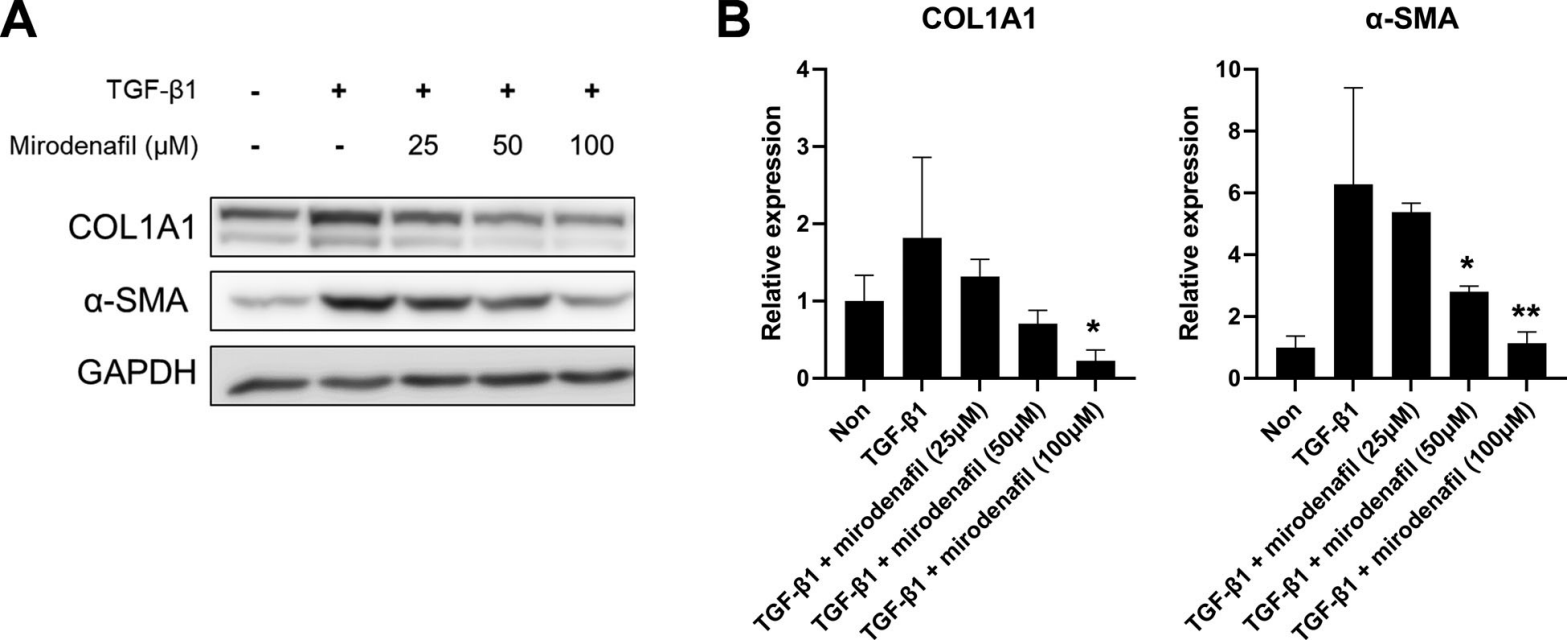
The anti-fibrotic effect of mirodenafil in human lung fibroblasts. (A) MRC-5 human fetal lung fibroblasts were treated with the indicated concentration of mirodenafil and human TGF-β1 (10 ng/ml) for 24 hr. The protein levels of COL1A1 and α-SMA were analyzed by western blot. (B) Data from western blots were quantified by densitometry. Data are presented as mean ± SD. *P < 0.05, **P < 0.01.

**Figure 6.**
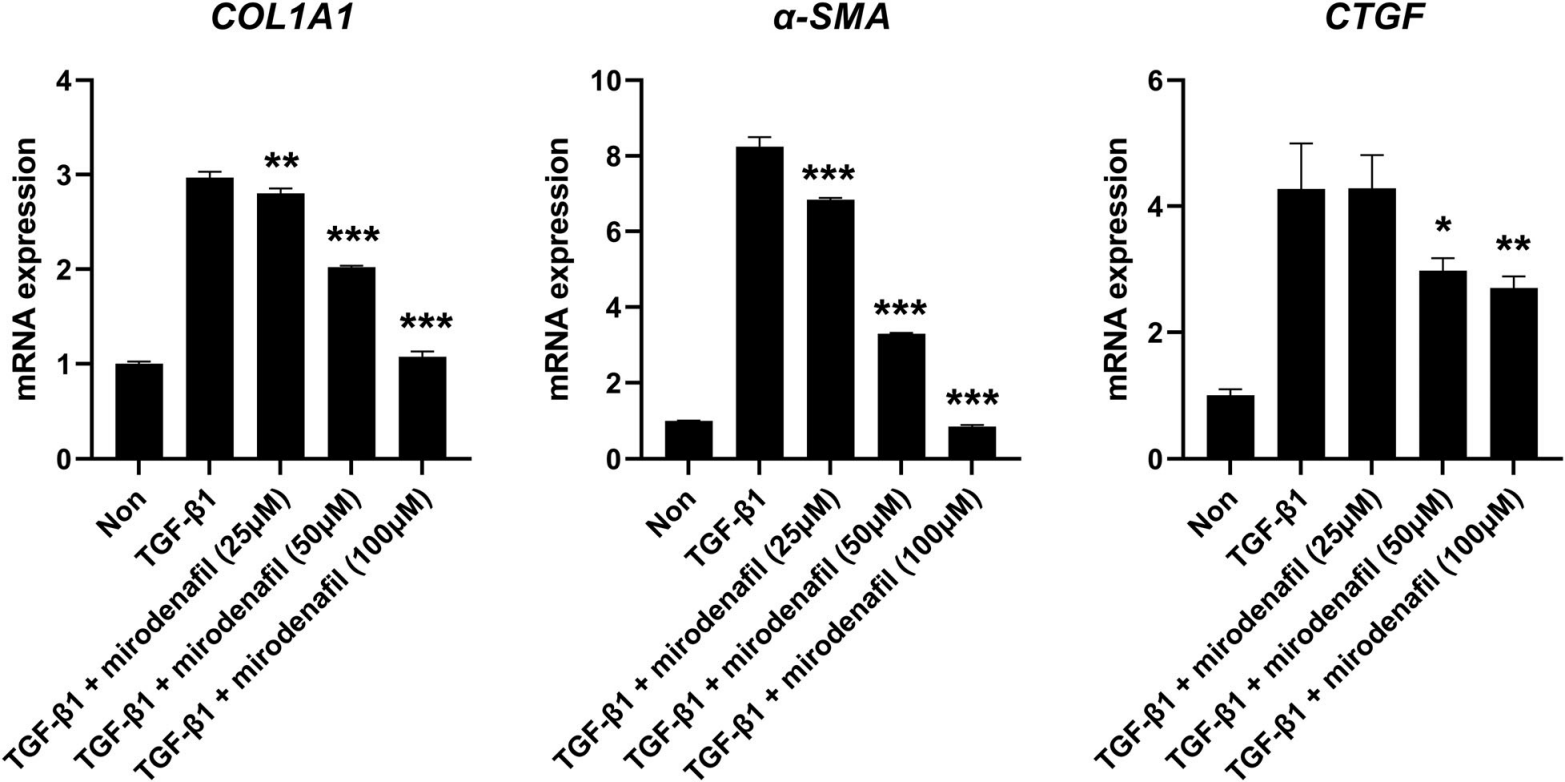
Mirodenafil inhibited the mRNA expression of the fibrosis marker in human lung fibroblasts. MRC-5 fibroblasts were treated with the indicated concentration of mirodenafil and human TGF-β1 (10 ng/ml) for 24 hr. The mRNA levels *of COL1A1, α-SMA*, and *CTGF* were assessed by qPCR. Expression data were normalized to *GAPDH* and presented as mean ± SD. *P < 0.05, **P < 0.01, ***P < 0.001.

We also examined the expression of fibrosis markers in MRC-5 human fetal lung fibroblasts. As expected, the increased protein levels of COL1A1 and α-SMA following treatment with TGF-β1 were also downregulated by mirodenafil in lung fibroblasts in a dose-dependent manner (Figure 5). In accordance with the results of NIH3T3, the mRNA expression of *COL1A1, α-SMA*, and *CTGF* were also induced by treatment with TGF-β1, and mirodenafil significantly reduced the expression of these profibrotic genes (Figure 6). These results suggested that mirodenafil may reduce the severity of both skin and lung fibrosis.

### Mirodenafil suppressed TGF-β/Smad signaling

**Figure 7.**
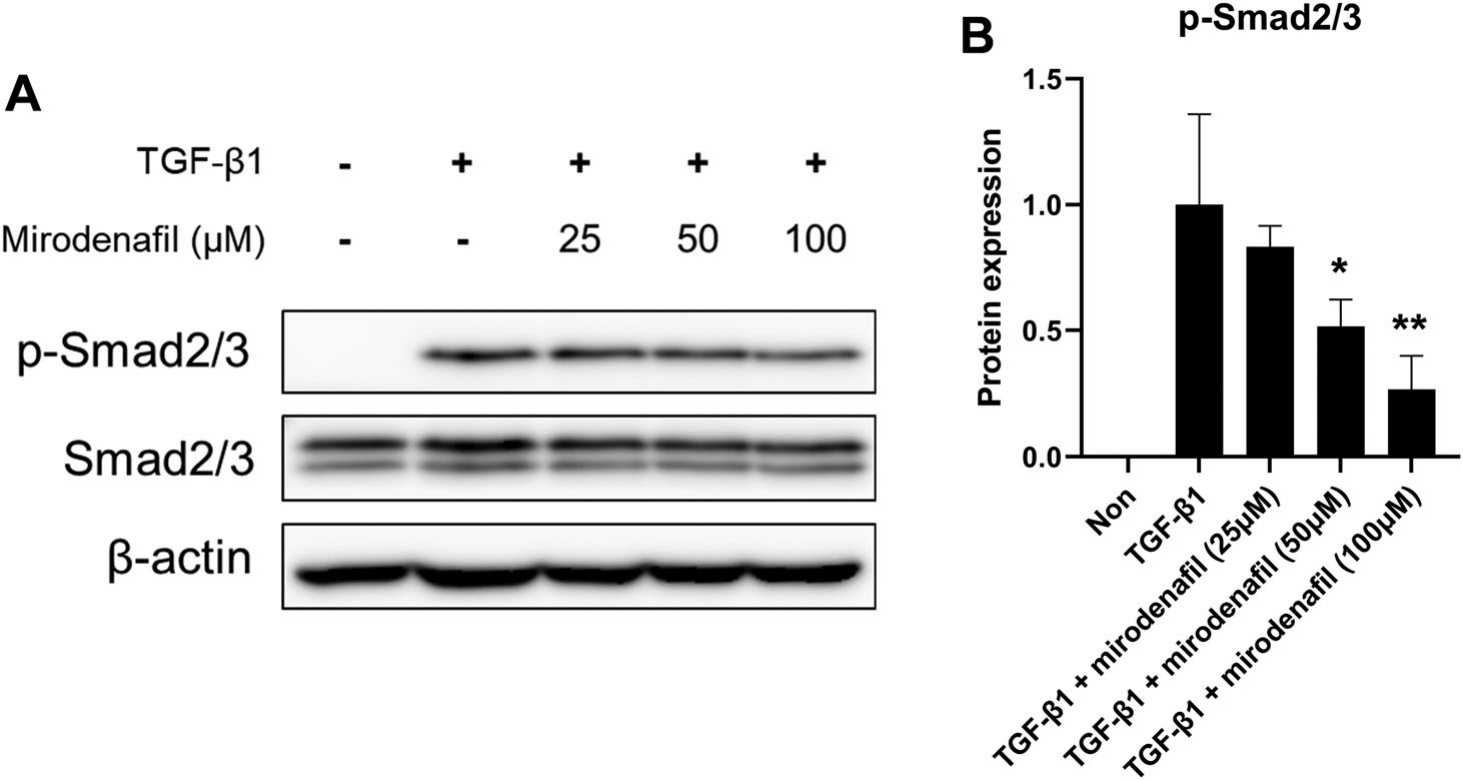
Mirodenafil inhibited TGF-β signaling. (A) NIH3T3 fibroblasts were treated with the indicated concentration of mirodenafil and mouse TGF-β1 (10 ng/ml) for 24 hr. The protein levels of p-Smad2/3 and Smad2/3 were analyzed by western blot. Beta-actin was used as loading control. (B) Data from western blots were quantified by densitometry. Data are presented as mean ± SD. *P < 0.05, **P < 0.01.

Since the TGF-β/Smad signaling pathway is critical for fibrosis in SSc, we also determined whether mirodenafil can regulate TGF-β/Smad signaling in fibroblasts. As expected, treatment with TGF-β1 induced the downstream activation of Smads, as indicated by the phosphorylation of Smad2/3. This was inhibited by mirodenafil in a dose-dependent manner, while the protein levels of Smad2/3 did not change (Figure 7). These results suggested that mirodenafil may ameliorate fibrosis by suppressing the TGF-β/Smad signaling pathway.

## Discussion

In the present study, we found that the PDE5 inhibitor mirodenafil alleviated skin fibrosis in the BLM-induced SSc model by suppressing the canonical TGF-β signaling pathway. It was also observed that the TGF-β-induced expression of COL1A1, α-SMA, and CTGF was decreased by mirodenafil both *in vivo* and *in vitro*. Taken together, our data suggested the potential therapeutic efficacy of mirodenafil for dermal fibrosis in SSc. In addition, given that mirodenafil increases cGMP by inhibiting PDE5, we also assumed that the cGMP-PDE5 pathway may play an important role in the fibrotic process in the pathogenesis of SSc.

TGF-β is a pleiotropic cytokine that regulates multiple physiological cellular processes, including immunity, angiogenesis, cellular proliferation, and apoptosis. After binding to its receptors, TGF-β plays a biological role through the phosphorylation of Smad 2/3 (canonical pathway) and activation of mitogen-activated protein kinases, phosphatidylinositide 3-kinase, or tumor necrosis factor receptor-associated factor 4/6 (non-canonical pathway). TGF-β is known to play a pivotal role in the fibrotic process in SSc by stimulating myofibroblasts, promoting perivascular inflammation, and activating immune cells (Asano 2020). Both canonical and non-canonical TGF-β pathways play important roles in the pathogenesis of SSc (Trojanowska 2009; Tsai et al. 2020). TGF-β can induce *Ctgf,* which can act as a downstream mediator of TGF-β, and both are important in the pathogenesis of fibrosis (Chen et al. 2016). TGF-β can also enhance the expression of *Col1a1* and *α-SMA*, which are responsible for myofibroblast activation (Chen et al. 2016). In addition, the development of skin fibrosis in the BLM-induced SSc model is largely dependent on the action of TGF-β. Therefore, TGF-β is considered a major therapeutic target for SSc. Considering that mirodenafil suppressed canonical TGF-β signaling and TGF-β-mediated expression of COL1A1, α-SMA, and CTGF in our study, we assumed that mirodenafil had potential therapeutic efficacy in the treatment of SSc.

cGMP, a unique messenger molecule produced in a variety of cells and tissues, targets numerous downstream signaling pathways, including PKG, and subsequently exhibits a wide range of biological actions (Friebe et al. 2020). The cGMP pathway is known to be actively involved in the pathogenesis of cardiovascular diseases, inflammation, neurodegeneration, and infectious disorders (Friebe et al. 2020). In addition, cGMP has been reported to inhibit fibrotic processes through various mechanisms. Beyer et al. demonstrated that increased cGMP by the use of a sGC stimulator blocked the non-canonical TGF-β signaling pathway and alleviated experimental fibrosis in an *in vitro* study (Beyer et al. 2012). Matei et al. reported that the sGC-cGMP-PKG pathway in fibroblasts of patients with SSc was significantly downregulated compared to that in healthy controls. Additionally, PKG, a downstream signaling enzyme of cGMP, was an important mediator of the anti-fibrotic property of SC stimulator (Matei et al. 2018). Similar to our findings, Higuchi et al. showed that increased intracellular cGMP by sildenafil, a PDE5 inhibitor, inhibited the TGF-β-induced expression of *COL1A1, COL1A2*, and *CTGF* in fibroblasts (Higuchi et al. 2015). Taken together, it is assumed that the cGMP pathway is closely linked with the pathogenesis of fibrosis and is considered an attractive therapeutic target for SSc.

Currently available Food Drug Administration (FDA)-approved PDE5 inhibitors include sildenafil, tadalafil, vardenafil, and avanafil; non-FDA approved PDE5 inhibitors include lodenfil, udenafil, and mirodenafil (Ahmed et al. 2021). PDE5 inhibitors prevent cGMP degradation by interfering with the binding of cGMP to the catalytic site of PDE5, consequently prolonging the action of the NO/cGMP signaling pathway. The accumulation of cGMP by PDE5 inhibitors decreases intracellular calcium levels, leading to vascular smooth muscle relaxation and vasodilatation. With this mechanism, PDE5 inhibitors are clinically approved for erectile dysfunction and PAH. In addition, the cGMP signaling pathway significantly contributes to the pathogenesis of vasculopathy in SSc. Moreover, PDE5 inhibitors have also been used for the treatment of vascular involvement in patients with SSc, such as PAH and Raynaud’s phenomenon. In addition to this notion, our findings suggest that mirodenafil also has potential anti-fibrotic properties, and cGMP may play an important role in the link between vasculopathy and fibrosis in SSc. Beyond its role in the stimulation of cGMP action, recent studies have demonstrated that PDE5 inhibitors decrease the activation of SSc fibroblasts by counteracting ROS effects and inhibiting the production of pro-inflammatory cytokines (Di Luigi et al. 2020; Antinozzi et al. 2021). Besides fibrosis, PDE5 inhibitors were also reported to have significant anti-inflammatory properties in various conditions such as muscle damage (Corinaldesi et al. 2021) and acute lung injury (Kosutova et al. 2018). Considering that aberrant autoimmune response is an important triggering factor for the development of SSc, PDE5 inhibition may ameliorate skin fibrosis through the suppression of inflammation. Therefore, the mechanisms by which PDE5 inhibitors ameliorate fibrosis in SSc seem to be diverse, and whether there are other mechanisms of action of PDE5 inhibitors require further research.

The limitation of our study is that we were unable to show the exact mechanisms by which mirodenafil inhibits TGF-β signaling and whether mirodenafil actually increased cGMP levels in our experimental system. Further research is needed to elucidate the mechanism by which mirodenafil regulates TGF-β signaling. However, despite this limitation, our data showed that mirodenafil has anti-fibrotic effects in an experimental animal model of SSc.

In conclusion, the present study revealed that mirodenafil ameliorated dermal fibrosis in a BLM-induced SSc model. The anti-fibrotic effect of mirodenafil was found to be mediated by the suppression of the canonical TGF-β signaling pathway. The TGF-β-mediated expression of COL1A1, α-SMA, and CTGF was also reduced by mirodenafil. We assumed that a link between the cGMP-PDE5 pathway and SSc fibrosis was corroborated in this study. Taken together, our findings may extend the understanding of the pathogenesis of SSc and provide evidence for the therapeutic potential of mirodenafil against the fibrotic process in SSc.
